# Enhanced CT-based radiomics model to predict natural killer cell infiltration and clinical prognosis in non-small cell lung cancer

**DOI:** 10.3389/fimmu.2023.1334886

**Published:** 2024-01-12

**Authors:** Xiangzhi Meng, Haijun Xu, Yicheng Liang, Mei Liang, Weijian Song, Boxuan Zhou, Jianwei Shi, Minjun Du, Yushun Gao

**Affiliations:** ^1^Department of Thoracic Surgery, National Cancer Center/National Clinical Research Center for Cancer/Cancer Hospital, Chinese Academy of Medical Sciences and Peking Union Medical College, Beijing, China; ^2^Department of Radiology, National Cancer Center/National Clinical Research Center for Cancer/Cancer Hospital, Chinese Academy of Medical Sciences and Peking Union Medical College, Beijing, China; ^3^Department of Thoracic Surgery, Sun Yat-sen Memorial Hospital, Sun Yat-sen University, Guangzhou, China

**Keywords:** radiomics, natural killer cell, infiltration, non-small cell lung cancer, prognosis, nomogram model, bioinformatic analysis

## Abstract

**Background:**

Natural killer (NK) cells are crucial for tumor prognosis; however, their role in non-small-cell lung cancer (NSCLC) remains unclear. The current detection methods for NSCLC are inefficient and costly. Therefore, radiomics represent a promising alternative.

**Methods:**

We analyzed the radiogenomics datasets to extract clinical, radiological, and transcriptome data. The effect of NK cells on the prognosis of NSCLC was assessed. Tumors were delineated using a 3D Slicer, and features were extracted using pyradiomics. A radiomics model was developed and validated using five-fold cross-validation. A nomogram model was constructed using the selected clinical variables and a radiomic score (RS). The CIBERSORTx database and gene set enrichment analysis were used to explore the correlations of NK cell infiltration and molecular mechanisms.

**Results:**

Higher infiltration of NK cells was correlated with better overall survival (OS) (*P =* 0.002). The radiomic model showed an area under the curve of 0.731, with 0.726 post-validation. The RS differed significantly between high and low infiltration of NK cells (*P <* 0.01). The nomogram, using RS and clinical variables, effectively predicted 3-year OS. NK cell infiltration was correlated with the ICOS and BTLA genes (*P <* 0.001) and macrophage M0/M2 levels. The key pathways included TNF-α signaling via NF-κB and Wnt/β-catenin signaling.

**Conclusions:**

Our radiomic model accurately predicted NK cell infiltration in NSCLC. Combined with clinical characteristics, it can predict the prognosis of patients with NSCLC. Bioinformatic analysis revealed the gene expression and pathways underlying NK cell infiltration in NSCLC.

## 1 Introduction

Lung cancer, which originates primarily from epithelial cells in the bronchial mucosa or alveoli, is the leading cause of cancer-related morbidity and is often accompanied by an unfavorable prognosis. Among its subtypes, non-small cell lung cancer (NSCLC) is predominant, comprising lung adenocarcinoma and squamous cell carcinoma (1). Although surgical resection remains the cornerstone of NSCLC management, its applicability diminishes in the advanced stages owing to the associated poor prognosis. For patients with advanced stages of NSCLC when surgery is not viable, therapeutic efforts focus on prolonging survival and increasing the quality of life, aiming for sustained survival despite the presence of tumor (2). Current prognostic markers for lung cancer include clinicopathological characteristics, laboratory diagnostic markers such as carcinoembryonic antigen and carbohydrate antigen 125 (3), and CT imaging modalities. However, these markers do not meet the evolving demands of precision medicine, underscoring the need to discover novel prognostic biomarkers that can guide personalized precision interventions.

Natural killer (NK) cells, pivotal for the body’s innate immune arsenal, possess the capability to eliminate target cells without antigen-specific stimulation and play instrumental roles in immune clearance and surveillance. Their role in targeting and eradicating tumor cells has been well established. For example, in hepatocellular carcinoma, curtailing antitumor sphingomyelin synthesis in peripheral NK cells can delineate alterations in the membrane structure of intratumoral NK cells, paving the way for innovative therapeutic strategies (4). Furthermore, in breast cancer, the interaction between HLA-G and the NK cell receptor KIR2DL4 amplifies the susceptibility of HER2-positive breast cancer cells to trastuzumab, elucidating the mechanisms underlying the resistance to trastuzumab (5). Studies have revealed that inhibition of IL6 can potentiate NK cell-mediated cytotoxicity in osimertinib-resistant EGFR-mutant NSCLC cells (6). Furthermore, another study demonstrated that the upregulation of SERPINB4 in NSCLC dampens NK cell-mediated cytotoxicity, suggesting potential therapeutic avenues for modulating immune responses against NSCLC (7). These findings emphasize the pivotal role of NK cells in orchestrating antitumor responses in NSCLC and other malignancies and highlight novel avenues for NK cell-centric therapeutic strategies.

Evaluation of NK cell infiltration is based predominantly on flow cytometry using fresh tissue specimens. Although effective, this technique has challenges, such as real-time detection constraints and elevated costs. Alternative methodologies include the use of public databases, such as ImmuCellAI and Cibersortx. However, these are contingent upon the availability of the corresponding genomic data and are susceptible to variables, such as specimen collection methods and antibody specificity. Imaging examinations continue to be a linchpin in clinical diagnostics because they are impervious to real-time variables, cost-effective, and widely accessible. This paved the way for radiomics, a discipline that uses medical imaging for research.

Recently, the integration of radiomics into medical research has increased. Radiomics creates models by extracting image features imperceptible to the human eye. When combined with clinical and pathological datasets, these models can help design diagnostic and prognostic frameworks. The spectrum of application of radiomics includes tumor diagnosis, post-therapeutic evaluations, and prognostic evaluations (8). Recent studies have demonstrated the versatility and effectiveness of CT-based radiomics in cancer diagnosis and treatment. One study (9) found that changes in CT numbers and tumor volume from cone-beam computed tomography can predict early response in NSCLC treatment, highlighting significant differences between responding and non-responding patients. Further, another study (10) showed that combining CT-based radiomics features with clinical factors can effectively predict PD-L1 and CD8+TILs expression levels in esophageal squamous cell carcinoma, thereby enhancing the accuracy of predictions. Similarly, a study (11) revealed that CT-based radiomics, when combined with clinical and morphological factors, can accurately predict PD-L1 expression levels and tumor mutation burden in advanced-stage NSCLC, which is crucial for guiding immunotherapy. Another research effort (12) demonstrated the potential of CT-based radiomics, in conjunction with clinical and radiological parameters, to effectively differentiate between immune checkpoint inhibitor-related pneumonitis and radiation pneumonitis in patients with advanced-stage NSCLC. These studies collectively underscore the growing importance of radiomics in oncological imaging and personalized medicine. In NSCLC, radiomic models enriched with multiregional attributes have been crucial in stratifying survival risks for patients diagnosed with clinical and pathological stage IA disease (13). A seminal study by Tong et al. (14) innovatively used machine learning algorithms to predict the expression levels of CD8 in tumor-infiltrating immune cells in NSCLC, leveraging PET/CT radiomics and clinical attributes, offering insights into the nuances of the tumor microenvironment. However, the radiomic domain has not yet been explored for the evaluation of NK cell infiltration in NSCLC.

Therefore, this study paved the way for the application of chest CT images in the construction of a radiomics model. The model is based on machine learning algorithms and aims to predict the abundance of NK cell infiltration in NSCLC tissues without invasive methods. By amalgamating bioinformatic analyses, our endeavor seeks to explore the molecular intricacies governing NK cell expression in NSCLC and their interaction with the immune microenvironment, which can provide novel perspectives for the diagnosis, therapeutic interventions, and prognostic evaluation of patients with NSCLC.

## 2 Materials and methods

### 2.1 Data acquisition

#### 2.1.1 Data analysis

**Figure 1:**
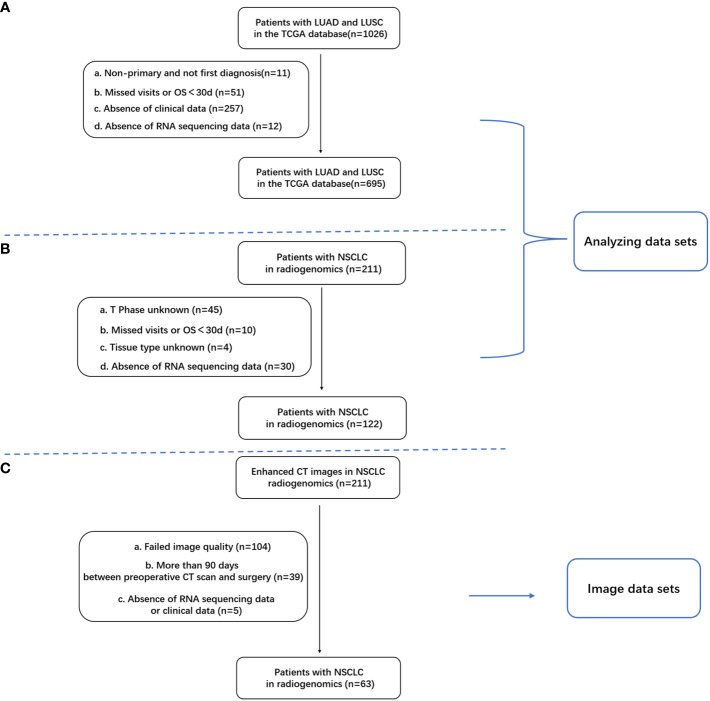
Inclusion and exclusion criteria for patients with NSCLC; **(A)** Criteria for patients with lung adenocarcinoma (LUAD) in the TCGA database; **(B)** 122 samples with clinical and RNA-seq data in the TCIA database; **(C)** Overlapping samples with clinical data, RNA-seq data, and enhanced CT images (n = 63).

Clinical, follow-up, and transcriptome sequencing data were sourced from the TCGA-LUAD and TCGA-LUSC datasets within The Cancer Genome Atlas (TCGA) database (https://portal.gdc.cancer.gov/) and the NSCLC radiogenomics dataset from The Cancer Imaging Archive (TCIA) database. The TCGA database contains 1,026 samples of lung adenocarcinoma and squamous carcinoma. After excluding samples with incomplete data (11 patients with nonprimary or initial diagnosis, 51 with absent follow-up data, 257 with missing clinical data, and 12 lacking RNA sequencing (RNA-seq) data), 695 samples were retained for the final analysis (**Figure 1**). The primary variable of interest was the number of NK cells. After applying the inclusion criteria, 122 samples from the NSCLC radiogenomics dataset were analyzed. The gene expression matrix for NSCLC was uploaded to the ImmuneCellAI database (http://bioinfo.life.hust.edu.cn/web/ImmuCellAI/), and immune cell infiltration was computed for each sample. NK cell infiltration data were categorized into high- and low-infiltration groups using the survminer package in R, with the low-infiltration group serving as a reference.

#### 2.1.2 Imaging dataset

Enhanced chest CT images were obtained from the NSCLC radiogenomics dataset within the TCIA database. Of the 211 samples in this dataset, 63 met the criteria for complete clinical data, RNA-seq data, and enhanced CT images (**Figure 1C**).

### 2.2 Correlation analysis of imaging histology

#### 2.2.1 Consistency assessment of enhanced chest CT images

The entire tumor region was delineated by two radiologists using the 3D Slicer software (version 4.10.2; https://www.slicer.org/) (15). Both radiologists independently described the lesions layer by layer, without knowledge of the patient’s clinical details or diagnostic results. The intraclass correlation coefficient (ICC) was used to gauge the consistency of the extracted imaging histological features based on the VOIs delineated by each radiologist. After one radiologist completed the outline for all cases, another radiologist randomly selected 10 samples using the “random number table method” to extract their imaging histological features. Typically, an ICC ≥0.75 indicated excellent agreement, 0.51–0.74 denoted moderate agreement, and <0.50 was deemed poor.

### 2.3 Radiomics feature screening

Feature extraction was performed using the open-source Python-based pyradiomics package for radiomic analysis of the shape, size, intensity, morphology, and texture; 107 radiomic features were extracted and data normalization was performed on the radiomics feature values. The LASSO algorithm and the stepwise regression algorithm using the R language “glmnet” package were used to filter the best subset of features.

### 2.4 Radiomic model construction

A logistic regression (LR) algorithm was used to construct models. The LR algorithm transforms linear regression through a sigmoid function such that the output value of the model is distributed between 0 and 1. The LR algorithm was fitted to the histological features using the glm function of the R language to build a binary classification model to predict the abundance of NK cell infiltration.

### 2.5 Radiomics model evaluation

**Figure 2:**
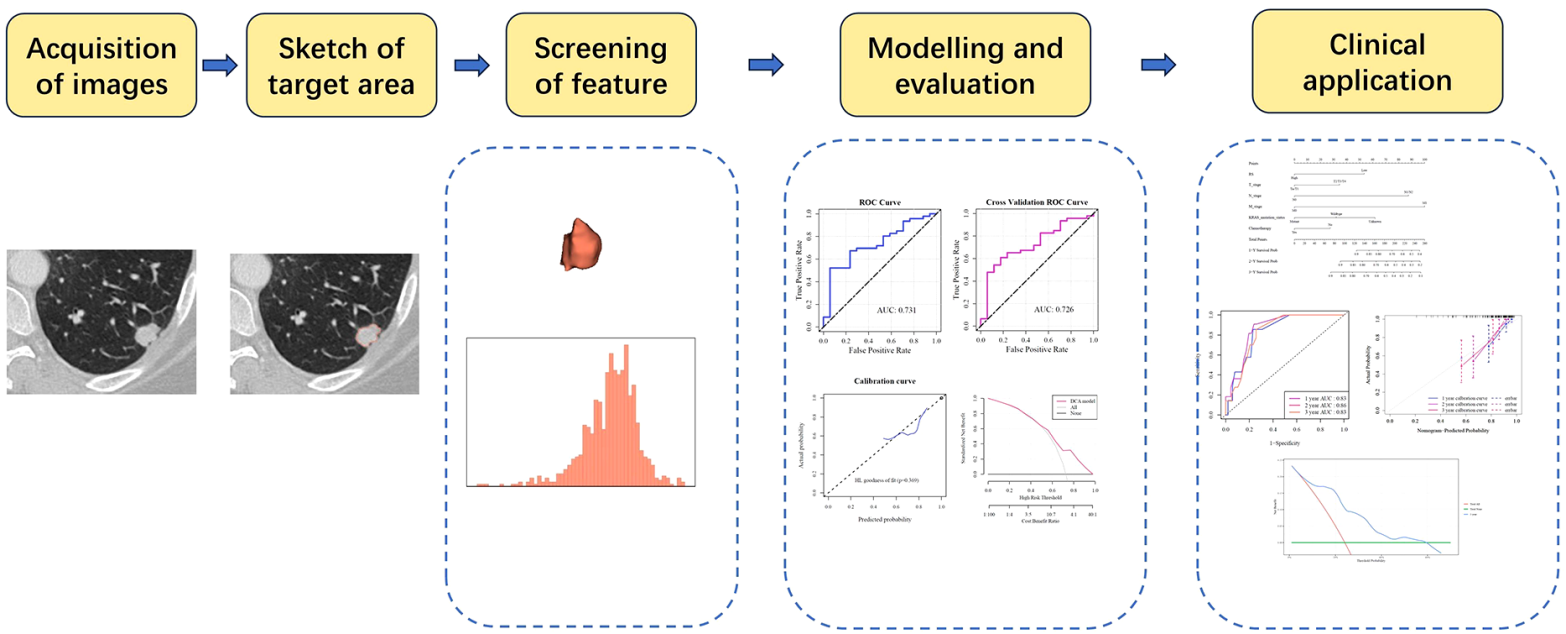
Flowchart of imaging histology analysis.

The efficacy of the LR radiomics model was evaluated with a five-fold internal cross-validation. Receiver operating characteristic (ROC) curves were used to evaluate the model’s accuracy (ACC), specificity (SPE), sensitivity (SEN), positive predictive value (PPV), and negative predictive value (NPV). Calibration of the imaging histology prediction model was evaluated by plotting a calibration curve (Hosmer–Lemeshow) goodness-of-fit test, and the clinical benefit of the imaging histology prediction model was demonstrated by plotting a decision curve. The Brier Score (BS) was used to evaluate the overall performance of the model. The radiomics model construction and evaluation process are illustrated in **Figure 2**.

### 2.6 Nomogram model construction and evaluation

Through stepwise regression screening of the clinical variables, the included variables were characterized using Akaike information criterion (AIC). The model was selected and constructed by choosing the smallest AIC statistic, and the column line graph nomograms of 1-, 2-, and 3-year survival probabilities of COX regression were plotted.

Radiomics score (Rad_score, RS) of the LR imaging histology model was merged with the clinical data to obtain the data of patients with NSCLC with RS, and then the cutoff value of RS was calculated using the survminer package. The patients were categorized into RS high-expression (n = 35) and low-expression (n = 28) groups. Finally, a nomogram prediction model was built using TCGA variables (T stage, N stage, M stage, KRAS mutation status, and chemotherapy) combined with the probability value (RS) predicted using the LR imaging genomics model. Time-dependent ROC, calibration, and decision curves were used for model evaluation.

### 2.7 Correlation analysis of survival factors

Kaplan–Meier survival curves were used to show changes in survival rate in different groups, in which the median survival time indicated the survival time corresponding to a survival rate of 50%. The R package “survival” was used to analyze the survival of each variable separately, and the R package “survminer” was used to summarize and visualize the results of the analysis. One-way and multifactorial COX regression analyses were performed using the R packages “survival” and “forestplot.” Prognostic risk factors were analyzed using univariate COX regression to explore the factors that influence overall survival (OS), and multivariate COX regression was used to explore whether a factor has an independent influence on OS and to explore the role of multiple factors.

### 2.8 Bioinformatics-related analysis

#### 2.8.1 Analysis of immune cell infiltration

The gene expression matrix of the lung cancer samples was uploaded to the CIBERSORTx database (https://cibersortx.stanford.edu/) and the immune cell infiltration of each sample was calculated. The correlation between the abundance of NK cell infiltration and the degree of immune cell infiltration was analyzed using the R package “corrplot.”.

#### 2.8.2 Gene set enrichment analysis

To investigate the molecular mechanism of expression differences between high and low NK cell immune infiltration abundances, the R package “clusterProfiler” was used to analyze the expression of hallmarks (h.all.v7.5.1. symbols.gmt), and KEGG (c2.cp.kegg. v7.5.1.symbols.gmt) gene sets were analyzed for GSEA enrichment.

### 2.9 Statistical analysis

The log-rank test was used to test the significance of survival between groups. The Wilcoxon test was used to compare whether the Rad_score differed between subgroups with high and low levels of cellular infiltration. Spearman’s rank correlation coefficient was used to correlate the abundance of immune infiltration by NK cells with the clinical characteristics of the tumors. *P <* 0.05 was considered significant.

## 3 Results

### 3.1 Correlation between NK cell immune infiltration and prognostic implications in the clinical characteristics of NSCLC

#### 3.1.1 Clinical characteristics correlation

From the TCGA dataset, 695 patients with lung cancer were included in the analysis. Using the R package “survminer,” a cutoff value of 0.124 was considered for NK cell infiltration. Based on this, the patients were stratified into high expression cohorts of NK high-expression (n = 287) and low-expression cohorts (n = 408). Significant distribution disparities were observed between the high and low NK cell infiltration groups in terms of histology (*P <* 0.001), sex (*P <* 0.001), and T stage (*P <* 0.001) (**Table 1**). The correlation heatmap indicated a pronounced association between the primary variable, the abundance of NK cell infiltration, and histology (*P <* 0.001), T stage (*P <* 0.001), and sex (*P <* 0.001) (**Supplementary Figure S1**).

**Table 1:** Association of NK cell expression with clinical features in TCGA.

Variables	Total (n = 695)	Low (n = 408)	High (n = 287)	P
Age, n (%)				0.338
** <66**	297 (43)	181 (44)	116 (40)	
** ≥66**	398 (57)	227 (56)	171 (60)	
Gender, n (%)				<0.001
** Female**	262 (38)	126 (31)	136 (47)	
** Male**	433 (62)	282 (69)	151 (53)	
Histology, n (%)				<0.001
** Adenocarcinoma**	320 (46)	162 (40)	158 (55)	
** Squamous cell carcinoma**	375 (54)	246 (60)	129 (45)	
T_stage, n (%)				<0.001
** T1**	184 (26)	84 (21)	100 (35)	
** T2**	404 (58)	250 (61)	154 (54)	
** T3/T4**	107 (15)	74 (18)	33 (11)	
N_stage, n (%)				0.846
** N0**	456 (66)	266 (65)	190 (66)	
** N1/N2/N3**	239 (34)	142 (35)	97 (34)	
M_stage, n (%)				0.326
** M0**	556 (80)	332 (81)	224 (78)	
** M1/MX**	139 (20)	76 (19)	63 (22)	
Chemotherapy, n (%)				0.841
** NO**	486 (70)	287 (70)	199 (69)	
** YES**	209 (30)	121 (30)	88 (31)	
Radiotherapy, n (%)				1
** NO**	629 (91)	369 (90)	260 (91)	
** YES**	66 (9)	39 (10)	27 (9)	
Residual_tumor, n (%)				0.855
** R0**	666 (96)	390 (96)	276 (96)	
** R1/R2**	29 (4)	18 (4)	11 (4)	
Smoking_status, n (%)				0.056
** Nonsmoker**	56 (8)	32 (8)	24 (8)	
** Current**	191 (27)	126 (31)	65 (23)	
** Former**	448 (64)	250 (61)	198 (69)	

From the NSCLC radiogenomics dataset in the TCIA database, an overlapping sample set with comprehensive clinical data, RNA-seq data, and enhanced CT images was obtained (n = 63). Using an NK cell infiltration abundance cutoff value of 0.0930, patients were categorized into a high-infiltration group (n = 46) and a low-infiltration group (n = 17) (**Table 2**).

**Table 2:** Association of NK cell expression with clinical features in TCIA.

Variables	Total (n = 63)	Low (n = 17)	High (n = 46)	P
Age, n (%)				0.116
**<66**	18 (29)	2 (12)	16 (35)	
**≥66**	45 (71)	15 (88)	30 (65)	
Gender, n (%)				0.333
** Female**	16 (25)	6 (35)	10 (22)	
** Male**	47 (75)	11 (65)	36 (78)	
Histology, n (%)				0.741
** Adenocarcinoma**	49 (78)	14 (82)	35 (76)	
** Squamous cell carcinoma**	14 (22)	3 (18)	11 (24)	
T_stage, n (%)				0.519
** Tis/T1**	31 (49)	10 (59)	21 (46)	
** T2/T3/T4**	32 (51)	7 (41)	25 (54)	
N_stage, n (%)				1
** N0**	51 (81)	14 (82)	37 (80)	
** N1/N2**	12 (19)	3 (18)	9 (20)	
M_stage, n (%)				1
** M0**	60 (95)	16 (94)	44 (96)	
** M1**	3 (5)	1 (6)	2 (4)	
Chemotherapy, n (%)				1
** No**	46 (73)	13 (76)	33 (72)	
** Yes**	17 (27)	4 (24)	13 (28)	
Radiotherapy, n (%)				1
** No**	57 (90)	16 (94)	41 (89)	
** Yes**	6 (10)	1 (6)	5 (11)	
KRAS_mutation_status, n (%)				0.24
** Mutant**	12 (19)	1 (6)	11 (24)	
** Unknown**	10 (16)	4 (24)	6 (13)	
** Wildtype**	41 (65)	12 (71)	29 (63)	
EGFR_mutation_status, n (%)				0.27
** Mutant**	13 (21)	5 (29)	8 (17)	
** Unknown**	10 (16)	4 (24)	6 (13)	
** Wildtype**	40 (63)	8 (47)	32 (70)	
Smoking_status, n (%)				0.098
** Nonsmoker**	9 (14)	5 (29)	4 (9)	
** Former**	36 (57)	7 (41)	29 (63)	
** Current**	18 (29)	5 (29)	13 (28)	

#### 3.1.2 Influence of NK cell immune infiltration abundance on NSCLC patient prognosis

**Figure 3:**
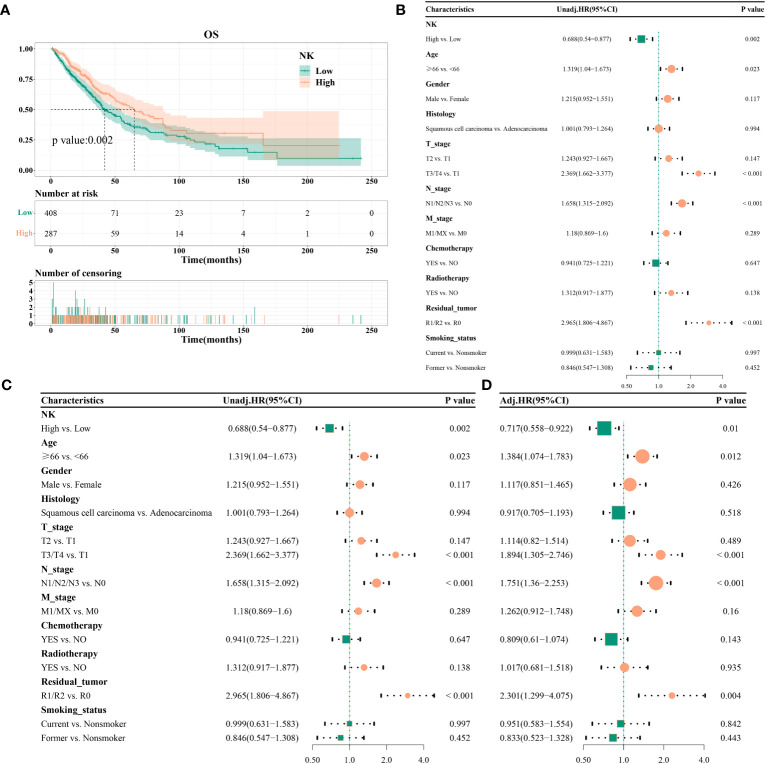
Effect of NK cell immune infiltration abundance on the prognosis of patients with non-small cell lung cancer; **(A)** KM curve, median survival time, and point-in-time survival rate; **(B)** univariate COX regression; **(C)** multivariate COX regression; **(D)** adjusted multivariate COX regression.

The high-infiltration group had a median survival time of 41.93 months, whereas the low-infiltration group had a median survival time of 65.1 months. Kaplan–Meier curves revealed a significant association of high NK cell infiltration abundance with better OS (*P =* 0.002) (**Figure 3A**). Univariate analysis identified high NK cell immune infiltration as a protective factor for OS (HR = 0.688, 95% CI 0.54–0.877, *P =* 0.002) (**Figure 3B**). Likewise, multifactorial analysis confirmed that high NK cell infiltration (HR = 0.717, 95%CI 0.558–0.922, *P =* 0.01) was a protective factor for OS (**Figure 3C**). After adjusting for multiple factors, age (*P =* 0.012), T stage (T3/T4 vs. T1, *P <* 0.001), N stage (*P <* 0.001), and tumor residue emerged as risk factors for OS (**Figure 3D**). Subgroup analysis indicated consistent effects of NK cell immune infiltration abundance on OS in different covariate subgroups (interaction test, *P >* 0.05) (**Supplementary Figure S2**).

### 3.2 Construction and evaluation of imaging histology-related models

#### 3.2.1 Screening and model construction of NK cell-based chest enhanced CT image histology features

**Figure 4:**
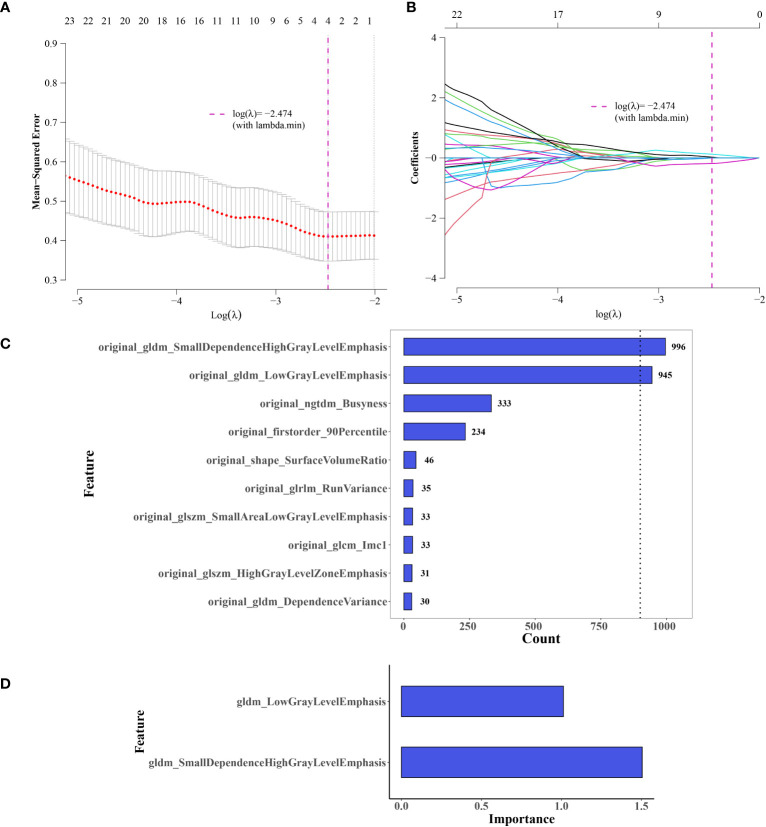
Selection of Imaging Histology Features and Model Development. **(A, B)** Feature selection using LASSO regression. **(C)** Selected features, two exceeding a value of 900. **(D)** Significance of the two retained features in the logistic regression (LR) model.

Feature selection using LASSO regression was shown in **Figures 4A, B**. After screening, two features with a frequency of occurrence >900 times were identified: original_gldm_SmallDependenceHighGrayLevelEmphasis and original_gldm_LowGrayLevelEmphasis (**Figure 4C**). The results of the consistency evaluation showed that the median ICC value of the radiomics features was 0.918, and there were 92 radiomics features with ICC values ≥0.75 (86% of all features). The ICC values of the two screened imaging histological features were >0.9 (**Figure 4D**). Imaging histology equation = original_gldm_SmallDependenceHighGrayLevelEmphasis × 0.615788 + original_gldm_LowGrayLevelEmphasis × (−0.3493 + 1.133413).

#### 3.2.2 Assessment of the radiomics model

**Figure 5:**
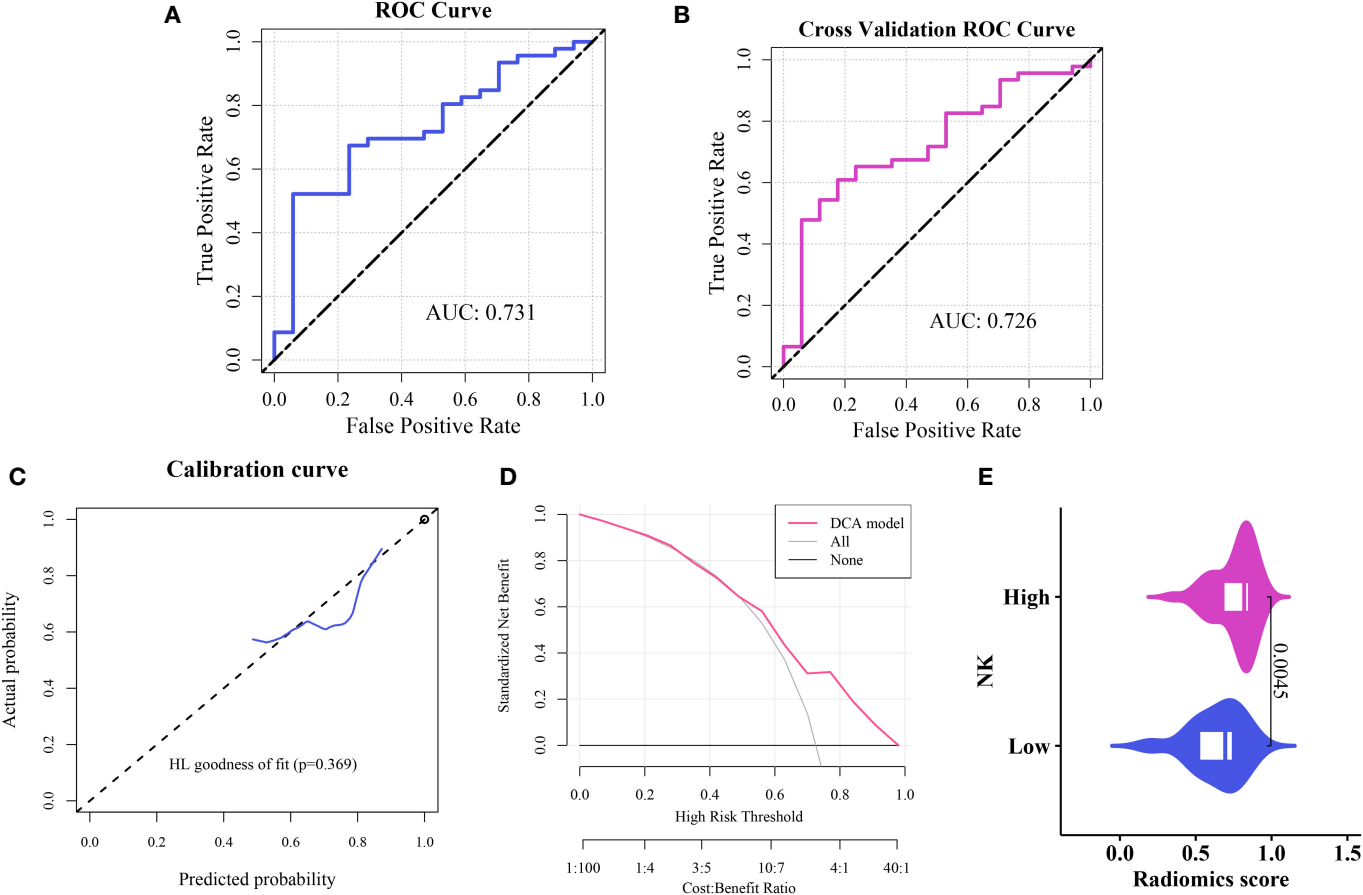
Construction and evaluation of the imaging histology model **(A)** ROC curve representation of the histological imaging model. **(B)** ROC curve after five-fold internal cross-validation. **(C)** Calibration curve of the model. **(D)** Hosmer–Lemeshow goodness-of-fit assessment. **(E)** Distribution of Rad scores between the high and low cellular infiltration groups.

The ROC curve indicated an area under the curve (AUC) of 0.731 for the model (**Figure 5A**), with an AUC of 0.726 (**Figure 5B**) after five-fold internal cross-validation. The calibration curve assessed using the Hosmer–Lemeshow goodness-of-fit test showed that the predictive probability of the radiomics model for high and low cellular infiltration abundance closely aligned with the actual outcomes (**Figure 5C**). Decision curve analysis revealed the substantial clinical utility of the model. For the training set, the threshold was set at 0.818, yielding an accuracy of 0.635, a sensitivity of 0.522, a specificity of 0.941, and a BS of 0.175. The five-fold cross-validation resulted in an accuracy of 0.667, a sensitivity of 0.609, a specificity of 0.824, and a BS of 0.184 (**Figure 5D**).

### 3.3 Radiomics model prediction and evaluation of NK cell infiltration abundance

We assessed the variability between the imaging histology of the LR model imaging. The output of the LR imaging histology model predicted the likelihood of NK cell infiltration, termed the Rad_score (RS). The findings indicated a notable difference in the RS distribution between the groups with high and low NK cell infiltration (*P <* 0.01); the group with elevated NK cell infiltration exhibited higher RS values. **Figure 5E** presents a visual representation.

### 3.4 Integrated model construction and clinical characteristics correlation assessment

**Figure 6:**
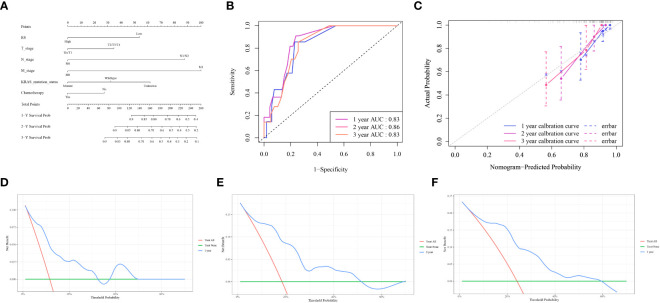
Nomogram model evaluation and clinical relevance **(A)** The comprehensive nomogram predictive model. **(B)** ROC curves illustrating the model’s predictive capacity for 1-, 2-, and 3-year overall survival (OS). **(C)** Calibration plot of the model. **(D–F)** Decision Curve Analysis (DCA) for 1,2,3-year OS, highlighting the high clinical utility of the model within the threshold range of 0.05–0.55.

To improve the prognostic capability of our model, we integrated clinically relevant factors. A nomogram predictive model was constructed using the RS with clinical variables (**Figure 6A**). Subsequent model evaluation revealed that the ROC curves demonstrated robust predictive efficacy for 1-, 2-, and 3-year OS (AUC values of 0.83, 0.86, and 0.83, respectively) (**Figure 6B**). The calibration plots for the model indicated that the curves for each time point were closely aligned diagonally, suggesting a minimal prediction error (**Figure 6C**). Furthermore, the decision curves revealed that the 1-, 2-, and 3-year OS for patients fell within a threshold range of 0.05–0.55, underscoring the high clinical utility of the model (**Figures 6D–F**).

### 3.5 Immune molecular mechanisms linked to NK cell infiltration in NSCLC

#### 3.5.1 Correlation between NK cell immune infiltration abundance and immune genes

**Figure 7:**
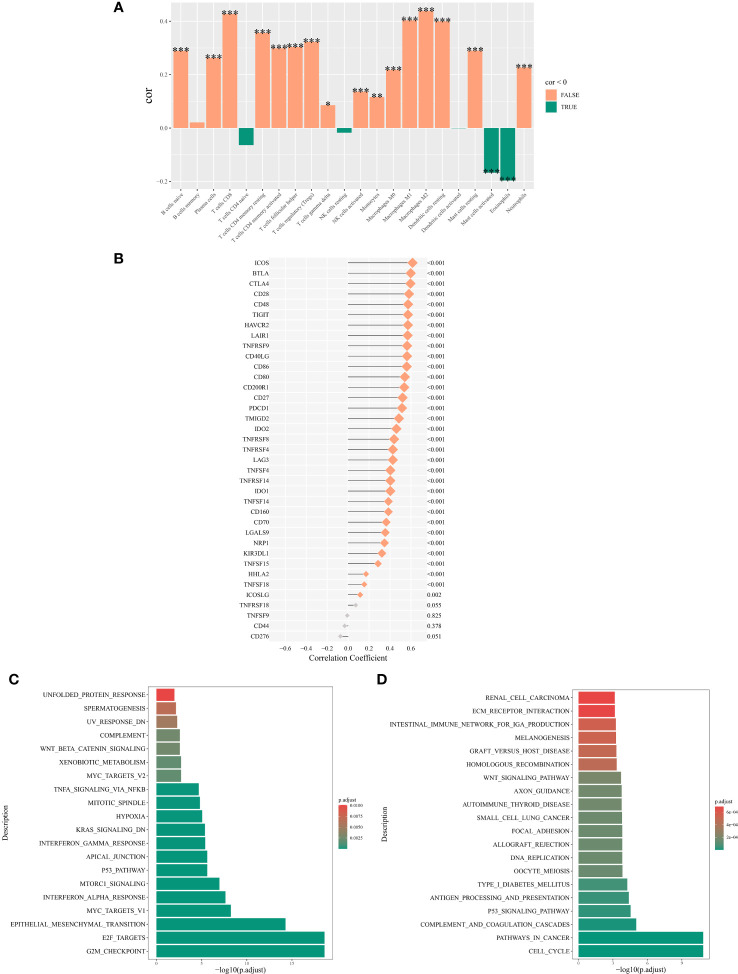
Mechanisms associated with the expression of NK cell immune infiltration abundance. **(A)** Correlation between NK cell immune infiltration abundance and correlation between NK cell immune infiltration abundance and immune cell abundance. **(B)** Correlation analysis of the main variable NK cell immune infiltration abundance and immune genes. **(C)** Top 20 significantly enriched pathways from the hallmark enrichment analysis results. **(D)** Top 20 significantly enriched pathways from the KEGG enrichment analysis results. *P < 0.05; **P < 0.01; ***P < 0.001.

Analysis using Spearman’s rank correlation coefficient revealed a positive association between the abundance of NK cell immune infiltration and immune genes (16). Specifically, the abundance of NK cell immune infiltration was significantly positively correlated with the ICOS and BTLA genes (*P <* 0.001) (**Figure 7B**). Furthermore, our examination of immune cell infiltration in lung cancer indicated that NK cell infiltration was positively associated with the infiltration levels of M0 and M2 macrophages (**Figure 7A**).

#### 3.5.2 Enrichment analysis of differentially expressed genes in NK cell immune infiltration subgroups

Among the hallmark gene sets, the GSEA highlighted the top 20 pathways. The genes differentially expressed between high and low NK cell immune infiltration subgroups were predominantly enriched in pathways such as TNF-α signaling via NF-κB, Wnt/β-catenin signaling, and p53 pathway (**Figure 7C**). In the context of the KEGG gene set, GSEA highlighted the 20 main pathways, with differentially expressed genes between the two subgroups significantly enriched in the p53 signaling pathway, Wnt signaling pathway, and cell cycel pathways (**Figure 7D**).

## 4 Discussion

Once metastasis occurs in NSCLC, with a 5-year relative survival rate of only 5%, the prognosis of NSCLC is poor (17, 18). Therefore, new prognostic predictors for the precise treatment of NSCLC are urgently needed. In this study, we analyzed the association between the histological characteristics of NSCLC and NK cells in a tumor microenvironment. This study aimed to predict NK cell infiltration in NSCLC noninvasively to improve prognosis prediction for patients. The predictive probability of the LR radiomics model for the abundance of NK cells agreed with the true value, and the model predictive value of RS positively correlated with the infiltration of NK cells. The nomogram model, which was established by combining clinical features (T, N, and M stages) with the RS of the radiomics model, showed a good predictive ability for patient prognosis.

In this study, to exclude the impact of clinical factors on the expression of NK cells and the prognosis of lung cancer, we employed a multivariate Cox regression analysis. After adjusting for the influence of 10 clinical demographic confounders, we found that NK cell expression remains a protective factor in the prognosis of lung cancer (OR: 0.717, 95% CI: 0.558-0.922, p=0.01). Additionally, through subgroup analysis and interaction tests, it was observed that there is no interaction between NK cell expression and the clinical demographic characteristics above in terms of the prognosis of lung cancer.

Several challenges remain in the contemporary field of NSCLC management. Despite the increased adoption of molecular targeted therapies and immunotherapies, a subset of patients, especially those with advanced disease stages, exhibit limited therapeutic responses (19). Despite their potential, the emergence of innovative diagnostic modalities faces barriers, such as high costs and intricate technical demands that limit their widespread adoption (20). Existing therapeutic regimens have yet to mitigate the associated adverse effects and complications, compromising patients’ quality of life.

NK cells play a role in many physiologic and pathologic processes in NSCLC. For example, NK cells in MHC class I-deficient lung adenocarcinoma displayed impaired cytotoxic activity towards tumor cells, associated with alterations in the expression of natural cytotoxicity receptors and ligands required for target cell recognition (21). NK cells produce cytokines, such as IFN-γ and TNF-α, which have anti-tumor effects. These cytokines can enhance the immune response by recruiting and activating other immune cells, including T cells, to the tumor site (22–24). NK cells interact with dendritic cells, macrophages, and T cells, modulating the immune response. And they can also shape the tumor microenvironment, influencing the efficacy of other immunotherapeutic strategies (25–27).

Recent advances in NSCLC research have highlighted the role of NK cells. These preliminary findings underscore the therapeutic potential of NK cells for NSCLC immunotherapy (28). To capitalize on the recognition of innate tumor antigens and cytotoxic capabilities of NK cells, ongoing research has aimed to improve their antitumor efficacy (29). Evidence suggests that specific methodologies can enhance NK cell activity and enhance tumor cell recognition (30). The intricate interplay between NK and lung cancer cells has emerged as a key research focus. Studies have shown that NK cells exert their cytotoxic effects by recognizing and engaging with specific receptors (31). The ability of the lung cancer microenvironment to modulate NK cell functionality and potentially attenuate its antitumor responses is a subject of intense scrutiny. Certain microenvironmental factors may impede NK cell functionality, diminishing their antitumor effects (32). Determining inhibitory mechanisms and strategizing countermeasures are critical research avenues.

In immunotherapeutic strategies, NK cells also play an important role. Many tumors, including NSCLC, exploit inhibitory pathways to escape NK cell-mediated killing (33, 34). New therapies aim to block these inhibitory signals (like KIRs, NKG2A) to enhance NK cell activity (35, 36). Strategies that boost activating signals on NK cells can enhance their ability to target NSCLC cells (37). This includes the use of antibodies that engage activating receptors like NKG2D or the use of cytokines like IL-15 to stimulate NK cell activity. NK cell activity and related markers are being explored as potential biomarkers to predict response to immunotherapy in NSCLC (38, 39).

In our study, we identified a positive association between NK cell infiltration and the expression of the ICOS (Inducible T-cell COStimulator) and BTLA (B and T Lymphocyte Attenuator) genes. A pronounced correlation was also observed between M0 and M2 macrophage infiltration. ICOS is a co-stimulatory molecule found on T cells. It plays a crucial role in T cell activation and survival. ICOS is part of the CD28 superfamily and interacts with its ligand, ICOSL, found on antigen-presenting cells (40–42). BTLA is an immune checkpoint molecule expressed on T cells, B cells, and to a lesser extent on NK cells and macrophages (43). It negatively regulates immune responses by binding to its ligand, HVEM (Herpesvirus Entry Mediator) (44–46).

ICOS and BTLA are associated with a variety of immune cells such as NK cells, M0 and M2 macrophages (47, 48). Firstly, NK cells typically do not express ICOS under normal physiological conditions. However, certain conditions, like chronic viral infections or certain cancer microenvironments, might induce ICOS expression on NK cells, potentially altering their function (49). NK cells can express BTLA. The engagement of BTLA on NK cells generally leads to an inhibitory signal, potentially reducing their cytotoxic function (50). The expression and functional impact of BTLA on NK cells can vary depending on the disease context and the microenvironment (50).

M0 Macrophages are considered to be in a resting state and can polarize into either M1 (pro-inflammatory) or M2 (anti-inflammatory) phenotypes based on the microenvironment (51, 52). M2 macrophages are associated with tissue repair, wound healing, and immune regulation (51). The role of ICOS and BTLA in M2 macrophages is less clear. However, given that BTLA is an inhibitory molecule, its expression in the macrophage context could be associated with the suppressive functions of M2 macrophages (53).

Gene enrichment analysis revealed that genes associated with NK cell immune infiltration were predominantly aligned with the p53 and Wnt signaling pathways. Thus, these insights will serve as a foundation for subsequent genomic and transcriptional regulation studies centered on NK cells.

By examining the gene expression profiles and signaling cascades of NK cells, we aim to elucidate the molecular dynamics of the participation of NK cells in NSCLC, informing the development of innovative therapeutic paradigms. In recent years, radiomics technology has become an important direction in NSCLC research. Radiomics analyzes medical imaging data using high-throughput methods that can reveal the microscopic and macroscopic features of tumors, thus contributing to a better understanding and treatment of NSCLC (54). First, histological imaging techniques have been effective in aiding the early diagnosis and staging of NSCLC (55). By analyzing specific imaging markers in CT or MRI, researchers can more accurately determine the size, location, and aggressiveness of a tumor, which can help develop more targeted treatment plans (56). Furthermore, histological imaging can predict the prognosis of a patient by analyzing the morphology and textural characteristics of the tumor. However, radiomics technology has certain shortcomings that limit its application in clinical practice (57). With the continuous development of multiomics technology, imaging histology technology is expected to further improve its application value in NSCLC research and treatment when combined with other bioinformatics methods. For example, by combining genomic and proteomic data, imaging genomics can provide a more comprehensive and in-depth tumor analysis, which can help to discover new therapeutic targets and strategies (56). Currently, there are no reports on radiomics and NK cells. Here, the radiomics model based on the features original_gldm_SmallDependenceHighGrayLevelEmphasis and original_gldm_LowGrayLevelEmphasis had a positive effect on NK cell infiltration in NSCLC and patient prognosis predictive effect, and the predicted probability of high and low NK cell infiltration abundance agreed with the true value. To the best of our knowledge, this is the first radiomics study to predict NK cell infiltration and patient prognosis in NSCLC. The present study fills a gap in imaging histology in NSCLC research and could facilitate the prediction of NK cell infiltration levels in NSCLC. The study also explored the genes, immune infiltration, and pathways associated with the abundance of NK cell infiltration by combining bioinformatics technology, which provides a new way to understand the molecular mechanism behind the imaging histology model in a more detailed and comprehensive manner.

Although the image histology model constructed in this study worked well, it had some shortcomings. First, data for this study were obtained from public databases with non-uniform image parameters. Second, the sample size was limited and needs to be further validated using a large-sample multicenter study. Finally, this study used a manual explanation of VOIs, which is also affected by subjective factors.

## 5 Conclusions

The radiomics model constructed in this study using machine learning algorithms based on enhanced CT images has good stability and diagnostic efficacy and can accurately predict the infiltration of NK cells in NSCLC. Furthermore, the comprehensive model established by combining relevant clinical characteristics can predict the prognosis of patients with NSCLC. Moreover, we explored the potential gene expression and regulation of the pathway underlying NK cell infiltration in NSCLC by combining bioinformatic technology, which provides a new strategy to assist in clinical decision-making and guide individualized precision diagnosis and treatment of NSCLC. In the near future, the combination of multicenter data and well-established mechanistic studies could present novel opportunities for the treatment of patients with NSCLC.

## Data availability statement

Publicly available datasets were analyzed in this study. This data can be found here: https://portal.gdc.cancer.gov/.

## Author contributions

XM: Conceptualization, Data curation, Validation, Writing – original draft. HX: Methodology, Writing – original draft. YL: Data curation, Writing – original draft. ML: Writing – review & editing. WS: Writing – review & editing. BZ: Writing – review & editing. JS: Writing – review & editing. MD: Writing – review & editing. YG: Investigation, Funding acquisition, Writing – review & editing.
