# Supplementary material for: Enhanced CT-based radiomics model to predict natural killer cell infiltration and clinical prognosis in non-small cell lung cancer
Source: Front Immunol. 2024 Jan 12;14:1334886. doi: 10.3389/fimmu.2023.1334886 (PMC10811188; doi:10.3389/fimmu.2023.1334886)
Supplement: Supplementary file 1 [file Table_1.docx]

Figure S1. Correlation heatmap showcasing the relationship between NK cell immune infiltration abundance and tumor clinical characteristics.

Figure S2 Subgroup analyses to analyze the effect of NK cell immune infiltration abundance on patient prognosis in different subgroups for each covariate
