# Supplementary figures and images for: Enhanced CT-based radiomics model to predict natural killer cell infiltration and clinical prognosis in non-small cell lung cancer
Source: Front Immunol. 2024 Jan 12;14:1334886. doi: 10.3389/fimmu.2023.1334886 (PMC10811188; doi:10.3389/fimmu.2023.1334886)

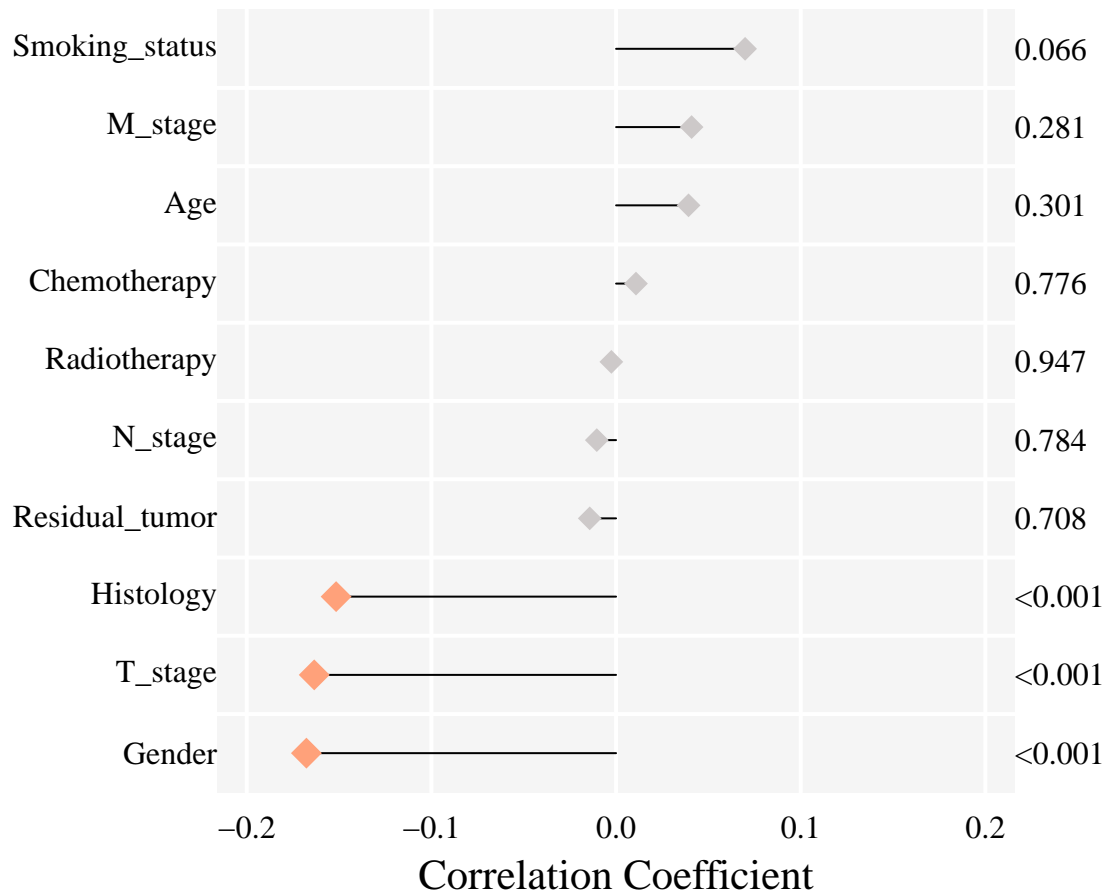

Supplement: Supplementary file 4 [file DataSheet_1.pdf]
