# Supplementary material for: Enhanced CT-based radiomics model to predict natural killer cell infiltration and clinical prognosis in non-small cell lung cancer
Source: Front Immunol. 2024 Jan 12;14:1334886. doi: 10.3389/fimmu.2023.1334886 (PMC10811188; doi:10.3389/fimmu.2023.1334886)

Characteristics	Unadj.HR(95%CI)	P value	HR(95%CI) for interaction	P for interaction
**Age**				
<66	0.886(0.604,1.3)	0.536	ref	
≥66	0.568(0.415,0.777)	<0.001	0.632(0.385,1.037)	0.069
**Gender**				
Female	0.573(0.382,0.86)	0.007	ref	
Male	0.791(0.582,1.076)	0.135	1.349(0.813,2.239)	0.246
**Histology**				
Adenocarcinoma	0.692(0.485,0.986)	0.042	ref	
Squamous cell carcinoma	0.685(0.489,0.959)	0.027	0.973(0.597,1.585)	0.911
**T_stage**				
T1	0.902(0.545,1.494)	0.689	ref	
T2	0.746(0.543,1.027)	0.073	0.823(0.454,1.493)	0.522
T3/T4	0.509(0.276,0.939)	0.031	0.546(0.247,1.205)	0.134
**N_stage**				
N0	0.666(0.481,0.923)	0.015	ref	
N1/N2/N3	0.684(0.476,0.983)	0.04	1.035(0.636,1.687)	0.889
**M_stage**				
M0	0.67(0.512,0.878)	0.004	ref	
M1/MX	0.813(0.462,1.43)	0.472	1.153(0.617,2.156)	0.655
**Chemotherapy**				
NO	0.782(0.589,1.038)	0.089	ref	
YES	0.467(0.291,0.751)	0.002	0.629(0.364,1.088)	0.098
**Radiotherapy**				
NO	0.686(0.53,0.888)	0.004	ref	
YES	0.716(0.354,1.447)	0.352	1.027(0.485,2.173)	0.945
**Residual_tumor**				
R0	0.71(0.554,0.911)	0.007	ref	
R1/R2	0.402(0.13,1.239)	0.112	0.48(0.152,1.514)	0.211
**Smoking_status**				
Nonsmoker	0.367(0.137,0.984)	0.046	ref	
Current	0.732(0.451,1.189)	0.207	1.305(0.495,3.444)	0.59
Former	0.697(0.516,0.943)	0.019	1.222(0.501,2.979)	0.66

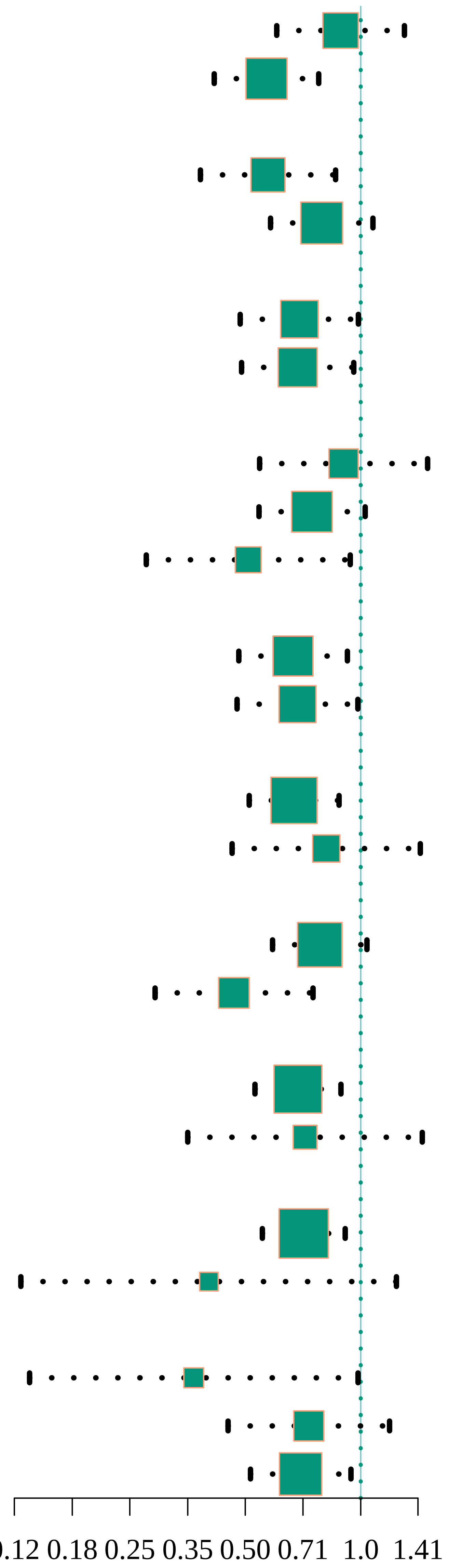

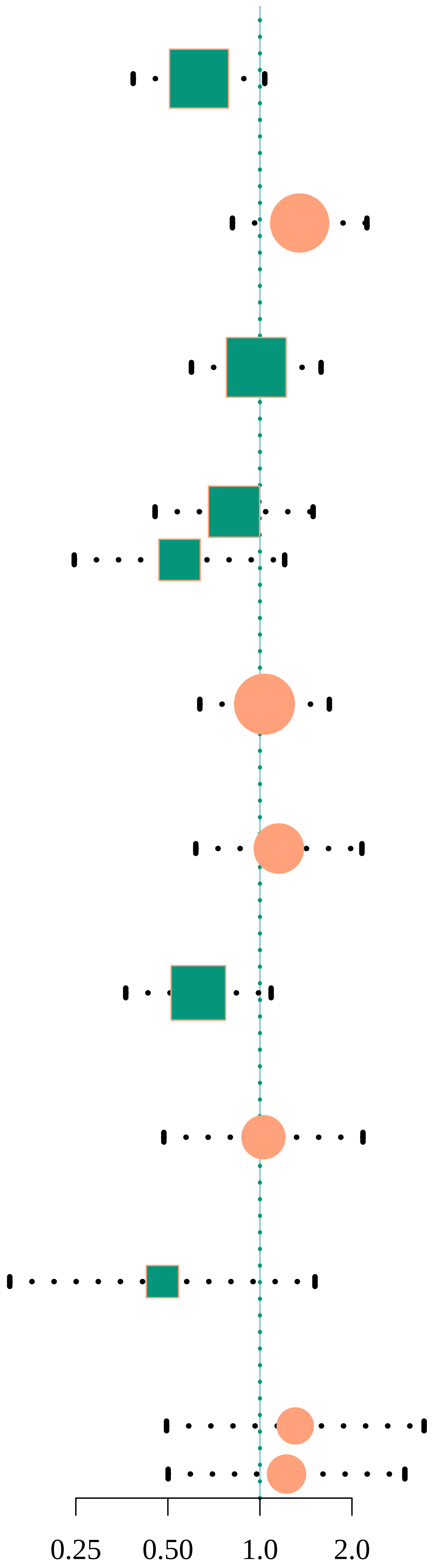

Supplement: Supplementary file 5 [file DataSheet_2.pdf]
